# Generation of pralatrexate resistant T‐cell lymphoma lines reveals two patterns of acquired drug resistance that is overcome with epigenetic modifiers

**DOI:** 10.1002/gcc.22884

**Published:** 2020-07-30

**Authors:** Luigi Scotto, Cristina Kinahan, Beatrice Casadei, Michael Mangone, Eugene Douglass, Vundavalli V. Murty, Enrica Marchi, Helen Ma, Changchun George, Francesca Montanari, Andrea Califano, Owen A. O'Connor

**Affiliations:** ^1^ Center for Lymphoid Malignancies Columbia University Medical Center New York New York USA; ^2^ Division of Experimental Therapeutics Columbia University Medical Center New York New York USA; ^3^ Department of Pathology and Cell Biology Columbia University Medical Center New York New York USA; ^4^ Department of Systems Biology Columbia University Medical Center New York New York USA

**Keywords:** pralatrexate, resistance, T‐cell lymphoma

## Abstract

While pralatrexate (PDX) has been successfully developed for the treatment of T‐cell lymphoma, the mechanistic basis for its T‐cell selectivity and acquired resistance remains elusive. In an effort to potentially identify synergistic combinations that might circumnavigate or delay acquired PDX resistance, we generated resistant cells lines over a broad concentration range. PDX‐resistant cell lines H9‐12 and H9‐200 were developed, each exhibiting an IC50 of 35 and over 1000 nM, respectively. These lines were established in vitro from parental H9 cells. Expression analysis of the proteins known to be important determinants of antifolate pharmacology revealed increase expression of dihydrofolate reductase (DHFR) due to gene amplification, and reduced folate carrier1 downregulation, as the putative mechanisms of resistance in H9‐12 and H9‐200 cells. Cross resistance was only seen with methotrexate but not with romidepsin, azacitidine (AZA), decitabine, gemcitabine, doxorubicin, or bortezomib. Resistance to PDX was reversed by pretreatment with hypomethylating agents in a concentration‐dependent fashion. Comparison of gene expression profiles of parental and resistant cell lines confirmed markedly different patterns of gene expression, and identified the dual specificity phosphatase four (DUSP4) as one of the molecular target of PDX activity. Reduced STAT5 phosphorylation following exposure to PDX was observed in the H9 but not in the H9‐12 and H9‐200 cells. These data suggest that combination with hypomethylating agents could be potent, and that DUSP4 and STAT5 could represent putative biomarkers of PDX activity.

## 1 INTRODUCTION

Folate metabolism plays a central role in multiple biological pathways and an unbalanced folate metabolism has been implicated in the pathogenesis of different diseases, including lymphomagenesis.^1^ Pralatrexate (PDX) was the first drug approved for patients with relapsed/refractory peripheral T‐cell lymphoma (PTCL). Unlike other folates, PDX was designed to have high affinity for the reduced folate carrier (RFC), which internalizes both natural forms of folic acid and antifols. This rapid and efficient internalization leads to relatively high intracellular concentrations. While PDX inhibits many of the same enzymes inhibited by other antifols like methotrexate (MTX), it is clear that its activity in PTCL goes well beyond the inhibition of these pathways. This is underscored by the fact that leucovorin does not mitigate its activity, which is the case for classical antifolates like MTX, and suggests that there are other targets the drug influences in PTCL that account for some component of its efficacy.^2^

Although PDX is significantly more cytotoxic when compared to first‐generation antifolates, its precise mechanism of action and T‐cell malignancy is not completely understood. PDX received regulatory approval for the treatment of relapsed or refractory PTCL in 2009. Early preclinical and clinical experiences have suggested an extraordinarily high level of activity in T‐cell malignancies, with minimal to no activity in patients with B‐cell lymphoid neoplasm or carcinomas.^3^, ^4^, ^5^ Despite the greater selectivity of PDX against T‐cell lymphomas, the overall response rate (ORR) in heavily treated patients is about 30%, though recent data from studies in Asia suggest ORR in the 50%.^6^, ^7^ Several lines of data suggest that PDX use earlier in the line of therapy can improve the ORR to about 45% to 50%, and lead to markedly better complete remission rates, and progression free and overall survival. This latter observation suggests that there is cross‐resistance with other conventional cytotoxic chemotherapy agents which accumulates over time.^8^

Intrinsic resistance to PDX appears to be overcome to some extent through the use of combinations, as preclinical and clinical data evaluating PDX combinations have shown marked synergy with histone deacetylase inhibitors (HDACi) like romidepsin,^9^ proteasome inhibitors like bortezomib,^10^ and cytidine analogs.^11^ For example, while the ORR to single agent PDX and romidespin are about 30% and 25%, respectively, the ORR to PDX plus romidepsin in a phase 1 study is in the 70% to 75% range, with a complete response rate of 29%.^9^, ^12^, ^13^

While the mechanistic basis for the PTCL selectivity and acquired resistance to PDX are still not entirely understood, it is clear the T‐cell malignancies represent one of those neoplastic diseases characterized by high rates of intrinsic drug resistance, and a penchant for rapid emergence of acquired resistance. Herein, we have developed a series of PDX‐resistant cell lines as a strategy to better understand mechanisms of acquired resistance and as a strategy to potential identify companion agents that might overcome mechanisms of intrinsic resistance.

## 2 MATERIALS AND METHODS

### 2.1 Materials

All drugs were purchased from Selleckchem (Houston, Texas) and were prepared in 100% DMSO.

### 2.2 Cell lines and culture

The cutaneous T‐cell lymphoma line H9 was obtained from the American Type Culture Collection (Manassas, Virginia). Parental and derived resistant cells were grown in RPMI 1640 medium with 10% fetal bovine serum.

### 2.3 Western blotting

Western blotting was performed according to standard protocols, using chemiluminescence detection system (Thermo Scientific, Waltham, Massachusetts). Primary antibodies against SLC19A1 (ThermoFisher, Waltham, Massachusetts), DHFR (Santa Cruz, Dallas, Texas), dual specificity phosphatase four (DUSP4), STAT5, phospho‐STAT5 and β actin (Cell Signaling, Danvers, Massachusetts).

### 2.4 Cytotoxicity assays

Cytotoxicity was performed on cultured cells using the Cell Titer Glo assay (Promega, Madison, Wisconsin) as previously described.^10^ Synergy of the combinations was calculated using Excess Over Bliss methodology.^10^ Luminescence was detected using the multimode plate reader GloMax Discover system (Promega, Madison, Wisconsin). For the pretreatment with AZA and decitabine, cell were exposed for 4 days to increased concentration of the hypomethylating agent (125, 250, and 500 nM for AZA and 6.25, 12.5, and 25 nM for decitabine). The compound was added to the cell culture every 24 hours for a 96‐hour period. Then, cells were washed and seeded at concentration of 3 × 10^5^ cells/mL and exposed to increased concentration of PDX.

### 2.5 Generation of PDX resistant cells

H9 cells were seeded at concentration of 3 × 10^5^ cells/mL into 75 cm^2^ culture flasks. To establish PDX resistance, the cells were initially incubated with low concentrations of PDX for 96 hours, then counted and reseeded. Concentrations of PDX were gradually increased and the process continued until the concentration of PDX in the medium reached between 30 and 200  nM after 160 days. Two H9‐derived cell lines were obtained that grew stably in medium containing PDX at concentration of 10 and 200 nM.

### 2.6 Quantitative reverse transcription polymerase chain reaction

RNA samples extracted from wild‐type and resistant cell lines were used for gene expression profiling and reverse transcription polymerase chain reaction (RT‐qPCR) analysis. cDNA was made using Omisncript RT Kit (Qiagen, Hilden, Germany). Taqman Fast Advanced Master Mix and FAM‐MGB primers were purchased from Thermo Fisher (Waltham, Massachusetts). RFC (Hs00953344_m1), folylpolyglutamate synthetase (FPGS) (Hs00191956_m1), DHFR (Hs00758822_m1), gamma‐glutamyl hydrolase (GGH) (Hs00914163_m1). Reactions were conducted on a StepOnePlus Real‐Time PCR System (Applied Biosystem, Foster City, California).

#### 2.6.1 Methylation specific PCR

Genomic DNA was extracted using a Quick‐DNA kit (Zymo Research, Irvine, California) and bisulfite treatment was carried out using a EZ DNA Methylation kit (Zymo Research, Irvine, California) following the manufacturer's instructions. Primers specific for the RFC promoter A and B regions and reaction condition were as previously described.^14^

### 2.7 Gene expression profiling

Total RNA was extracted using RNeasy mini kit (Qiagen, Hilden, Germany) from cells collected after 24 hours incubation with or without drugs (MTX and PDX) and RNA quantitation and quality over assessed by the Agilent Bioanalyzer 2100. RNA libraries prepared from poly‐A pull‐down enrich mRNAs (Illumina TruSeq RNA prep kit, San Diego, California), were sequenced at the Columbia Genome Center using Illumina HiSeq2500/HiSeq4000. DEseq software, an R package based on a negative binomial distribution that models the number reads from RNA‐seq experiments and test for differential expression, was employed to test for differentially expressed genes under various conditions. For visualization, raw counts were normalized sample‐wise to reads per million and differential expression was calculated for each cell line as a z‐score centered at untreated controls. Hierarchical clustering was calculated by euclidean distance using the hclust function in the stats R package and visualized using the heatmap.2 function within the gplots R package. Unclustered heat maps were also generated with the heatmap.2 function with samples organized by cell line and drug concentrations and genes organized by pathway annotation. Principal component analysis (PCA) was prcomp function in the stats R package. In the matrix, each column represents a sample and each row represents a gene.

## 3 RESULT

### 3.1 Differential H9, H9‐12, and H9‐200 cells sensitivity to folate antagonist

**FIGURE 1:**
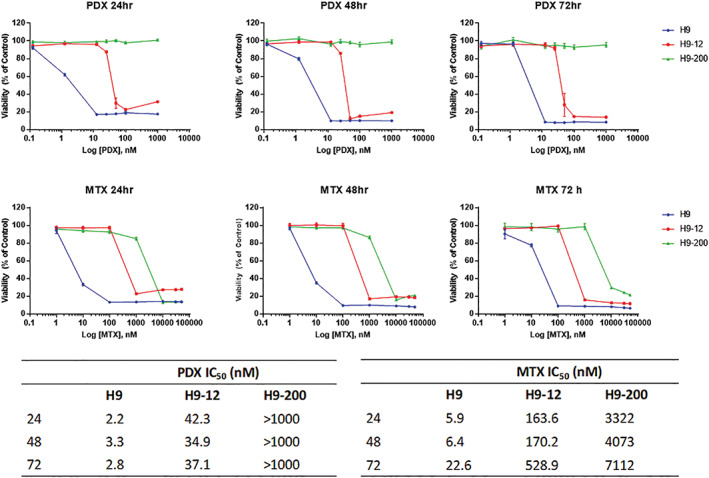
H9, H9‐12, and H9‐200 differential sensitivity to pralatrexate (PDX) and methotrexate (MTX). Growth inhibition curves following 24, 48, and 72 hours exposure of H9 (blue), H9‐12 (red), and H9‐200 (green) cells to increased PDX or MTX concentrations. IC_50_ values given in nanomolar concentrations for each time point. Error bars represent the SD of three or more separated experiments [Color figure can be viewed at wileyonlinelibrary.com]

Two drug resistant cell lines, named H9‐12, and H9‐200, were derived from exponentially growing H9 cells, following exposure to increased PDX concentrations. H9‐12 and H9‐200 cells showed no sensitivity to drug concentrations that clearly induce cell death in the H9 parental cell line, indicating the emergence of a resistant phenotype. Cell viability of H9‐12 cells was not affected by a 20‐fold increase in drug concentration relative to H9 parental cells while H9‐200 cells showed no sensitivity to all tested PDX concentrations (≥1 mM). Figure 1 shows the sensitivity of H9, H9‐12, and H9‐200 cell lines to increasing concentrations of both PDX and MTX. All cell lines exhibited a concentration‐dependent resistance to PDX and MTX, though PDX retains about 1‐log greater potency than MTX across the three cells lines.

A significant fold increase was observed in the concentration of PDX and MTX required to affect cell viability by 50% in drug resistant cells relative to their corresponding parental cells. The IC50 for PDX and MTX, after 48 hours of drug exposure, were estimated to be 3.3 and 6.4 nM for H9 cells, 34 and 170.2 nM for H9‐12 cells, and >1000 and 4073 nM for H9‐200 cells, respectively (Figure 1 and Supporting information Figure 1).

In order to evaluate the stability of the drug resistance phenotype, the H9‐12 and H9‐200 cell lines, grown for over a month in absence of drug, were reexamined for their relative sensitivity to increase concentration of PDX. H9‐12 and H9‐200 cells, grown in absence of drug, still demonstrated resistance to PDX when compared to the H9 parental cell line. The IC50 for PDX was 14.17 nM for H9‐12 and >1000 nM for H9‐200 (Supporting information Figure 1).

### 3.2 H9, H9‐12, and H9‐200 display similar sensitivity to HDACi, AZA, decitabine, doxorubicin, gemcitabine, and bortezomib

**FIGURE 2:**
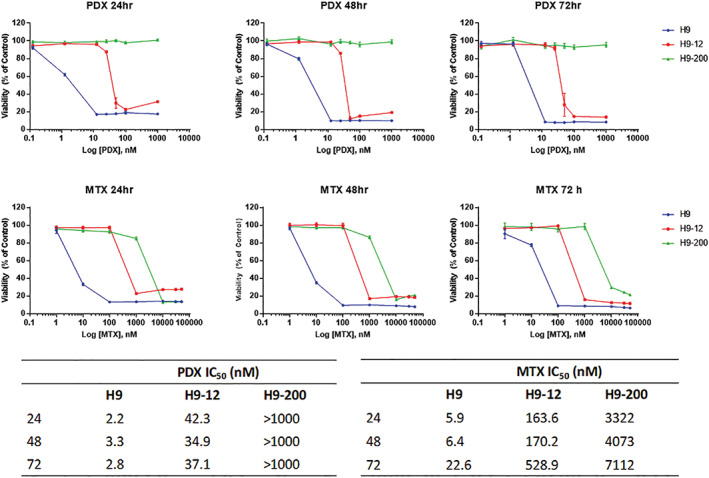
H9, H9‐12, and H9‐200 similar sensitivity to romidepsin, azacitidine, and decitabine. Growth inhibition curves following 24 (blue), 48 (red), and 72 (green) hours exposure to increased drug (Romidepsin, Azacitidine, and Decitabine) concentrations. IC_50_ values given in nanomolar and micromolar concentration for each time point. Error bars represent the SD of three or more separated experiments [Color figure can be viewed at wileyonlinelibrary.com]

To evaluate the patterns of resistance to other drugs, H9, H9‐12, and H9‐200 were exposed to increased concentrations of the HDACi romidepsin, two hypomethylating agents (AZA and decitabine), an anthracycline (doxorubicin), the synthetic pyrimidine nucleoside prodrug gemcitabine and the proteasome inhibitor bortezomib. Concentration and time dependent sensitivity of the parental and the two resistant cell lines to all six single agents were similar (Figure 2 and Supporting information Figure 2). The estimated IC50s at 48 hours from exposure were 3.1 nM for romidepsin, 0.9 μM for AZA, >100 μM for decitabine, 168.7 nM for doxorubicin, 5.9 nM for gemcitabine, and 4.3 nM for bortezomib.

### 3.3 Differential expression of DHFR, FPGS, GGH, and RFC in the H9, H9‐12, and H9‐200 cells

**FIGURE 3:**
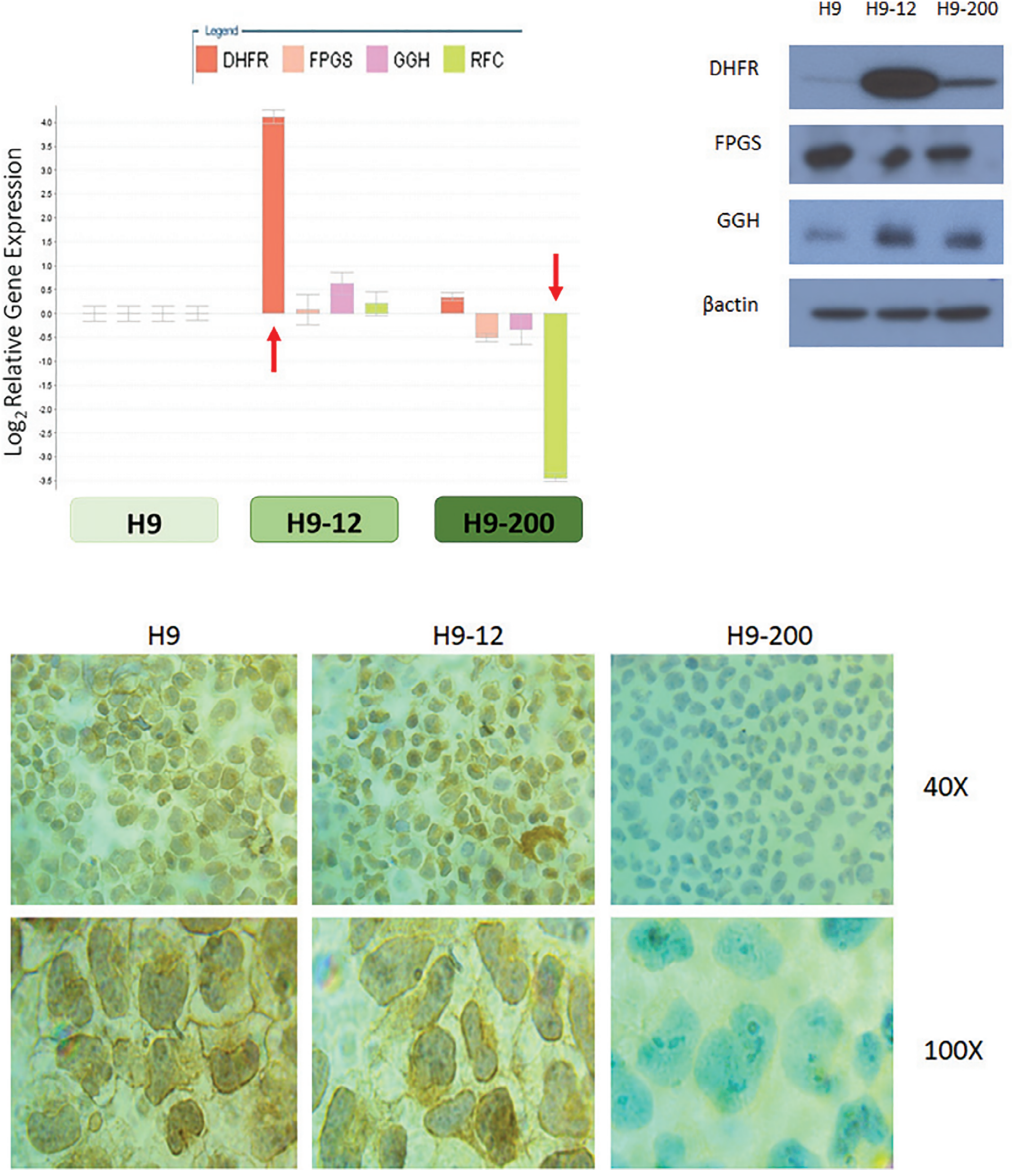
Gene expression of folate pathway genes in H9, H9‐12, and H9‐200 cell lines. A, Dihydrofolate reductase (*DHFR)*, folylpolyglutamate synthetase (*FPGS)*, gamma‐glutamyl hydrolase (*GGH)*, and reduced folate carrier *(RFC)* mRNA levels in H9‐12 and H9‐200 cells determined by quantitative real‐time RT‐PCR as compared to H9 parental cells. Arrows indicate *DHFR* and *RFC* RNA levels. B, DHFR, FPGS and GGH protein levels in H9, H9‐12, and H9‐200 cells as determined by Western blot analysis. C, Immunohystochemistry analysis of RFC expression in H9, H9‐12, and H9‐200 [Color figure can be viewed at wileyonlinelibrary.com]

Mechanisms of resistance to folate antagonists has been correlated to deregulated expression of DHFR, GGH, FPGS, and RFC.^12^, ^15^, ^16^, ^17^, ^18^, ^19^, ^20^ In order to identify those traditional molecular mechanisms that would explain the patterns of antifol resistance we quantitated the expression of the *DHFR*, *FPGS*, *GGH*, and *RFC* genes in the H9 parental cell line and the two PDX resistant cell lines H9‐12 and H9‐200 as determined through RT‐qPCR, western blot analysis and immunocytochemistry (IHC). As shown in Figure 3A, when compared to the parental H9 cells, a substantial increase (5‐fold) in RNA levels for the *DHFR* gene was identified in the H9‐12 cell line. Increase (0.5‐fold) in *DHFR* RNA levels was also observed in the H9‐200 cells though of a lesser extent. Western blot analysis confirmed a corresponding increase in DHFR protein levels in the H9‐12 and H9‐200 cells (Figure 3B). Increased GGH and reduced FPGS protein levels were also identified in H9‐12 and H9‐200 cells when compared to parental H9 cells, but without a clear relationship with the observed RNA levels. Finally, a substantial decrease in RNA levels for the *RFC* gene was observed in the H9‐200 cells compared to H9 and H9‐12 cells (Figure 3A). IHC analysis of the RFC protein expression in parental and drug resistant cell lines, correlated with the differential expression observed at the RNA level (Figure 3C).

These data suggest that the molecular mechanism mainly responsible for PDX/MTX resistance in the H9‐12 cells is the over expression of the *DHFR* gene, while in the H9‐200 cells, the principle mechanism of resistance was reduced *RFC* expression. Thus, two distinct concentration dependent patterns of PDX resistance. Based on these findings, we sought to determine the genetic basis for the resistance.

### 3.4 *DHFR* gene amplification and MSP‐PCR analysis of *RFC* promoter

**FIGURE 4:**
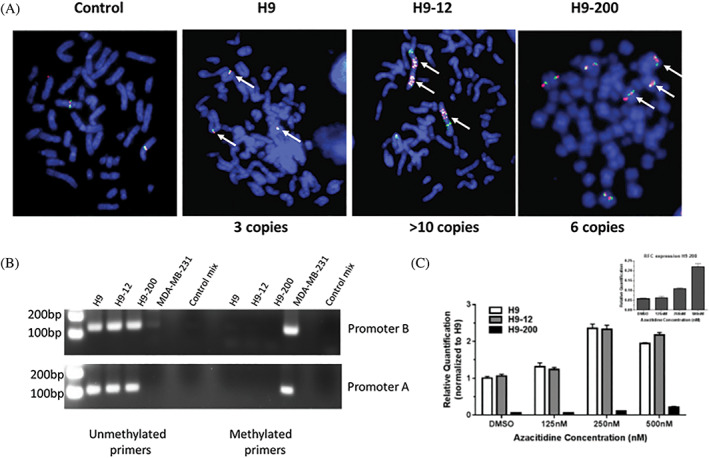
Dihydrofolate reductase (*DHFR)* gene amplification and MS‐PCR analysis of reduced folate carrier *(RFC)* promoter region. A, *DHFR* cytogenetic analysis of H9, H9‐12, and H9‐200 cells using two colors fluorescence in situ hybridization analysis. Arrows indicate signals relative to amplified *DHFR* gene in metaphase chromosome spreads. B, *RFC* promoter methylation specific PCR in H9, H9‐12, H9‐200, and MDA‐231 cells. C, *RFC* mRNA level quantitative analysis in H9, H9‐12, and H9‐200 cells exposed to increased concentration of azacytidine (AZA) for 96 hours. Samples were normalized to *RFC* mRNA level in DMSO treated H9 cells. Bars represent SD of three determinations. For all the analyses similar results were obtained in three independent determinations. Top right histogram shows an enhanced representation of the induced RFC mRNA expression by azacitidine in the H9‐200 cells. DMSO, dimethyl sulfoxide; MS‐PCR, methylation specific polymerase chain reaction [Color figure can be viewed at wileyonlinelibrary.com]

As has been shown by the seminal work of Bertino and coworkers,^19^, ^21^, ^22^ resistance to MTX has been attributed to increased expression of *DHFR* through *DHFR* gene amplification. As shown in Figure 4A, cytogenetic analysis of H9, H9‐12, and H9‐200 using fluorescence in situ hybridization analysis with a two color DHFR fluorescent probe established the presence of 2, 10 and 4 copies of the *DHFR* gene, respectively. These data confirm that the increased expression of the *DHFR* gene in the H9‐12 and H9‐200 cell lines was the result of gene amplification.

Epigenetic dysregulation can also play an important role in drug resistance, by silencing genes controlling the major determinants of antifol sensistivity and resistance. Methylation of the *RFC* promoter has been reported as the underlying mechanism for the antifolate resistance observed in colorectal cancer,^23^ systemic lupus erythematosus,^24^ the MTX resistant cell line M805, established from a patient with malignant fibrohistocytoma^14^ and the breast cancer cell line MDA‐MB‐231, where a 1400 bp *RFC* promoter region was identified as a CpG island and found methylated.^14^, ^25^ We explored the methylation status of the *RFC* promoter in the H9, H9‐12, and H9‐200 cell lines via methylation specific PCR, using as positive control the genomic DNA extracted from the MDA‐MB‐231 cell line. As shown in Figure 4B, neither promoter region A nor B shows any evidence of methylation at genomic level in H9, H9‐12, and H9‐200 cells when compared to MDA‐231 cells. This result excludes the likelihood that reduced expression of the *RFC* gene in the H9‐200 cells is due to promoter methylation. Rothem et al^26^ have reported that reduced expression of transcription factors as CREB1, ATF1, USF1, FOS, JUN, Sp3, and Sp1 is responsible for a major decrease in *RFC* mRNA expression in the CCRF‐CEM and derived human leukemia cell lines. Hence, we then explored any potential relationship between the expression of these transcription factors and the reduced *RFC* seen in H9‐200. Western blot analysis of H9, H9‐12, and H9‐200 cells showed a similar level of expression for the transcription factor USF1 in all three cell lines, but a reduced level of expression of the transcription factors Sp1, Sp3 and JUN in H9‐12 and H9‐200 when compared to parental H9 cells (Supporting information Figure 3). H9‐12 cells do not show decreased *RFC* expression, thus taking in consideration the differential expression of RFC in H9‐12 and H9‐200 we can exclude that the decrease expression of the RFC gene in H9‐200 is the result of a reduced availability of the Sp3 and JUN transcription factors.

Although promoter methylation was not the reason for the lower *RFC* expression, we explored the potential of global genome wide methylation on the lower levels of RFC in H9‐200. We treated cells with 5‐azacytidine (AZA) and decitabine (DAC) in order to determine if these hypomethylating agents could restore *RFC* expression and consequently sensitivity to PDX. As first step, H9, H9‐12, and H9‐200 were exposed to increased concentrations of AZA and DAC. Concentration and time dependent sensitivity of the parental and the two resistant cell lines to the two single agents were similar (Figure 1), again confirming minimal to no cross‐resistance to these drugs. The estimated IC50s at 48 hours from exposure were 0.9 μM for AZA and >100 μM for DAC. As we and others have demonstrated in the past, maximal demethylation requires continuous exposure to the hypometylating agent.^27^ Continuous exposure to AZA with 24 hours interval replacement demonstrated that H9, H9‐12, and H9‐200 cells demonstrated a 2‐ to 4‐fold increase in *RFC* mRNA level as determined by qPCR analysis (Figure 4C). A 2‐fold increase in *RFC* mRNA level was observed in H9 and H9‐12 cells while a 4‐fold increase was seen in the H9‐200 cells (Figure 4C). However, the *RFC* mRNA level in the H9‐200 exposed to AZA was substantially reduced when compared to H9 and H9‐12 mRNA levels.

### 3.5 Pretreatment with hypomethylating agents restore PDX sensitivity in H9‐200 cells

**FIGURE 5:**
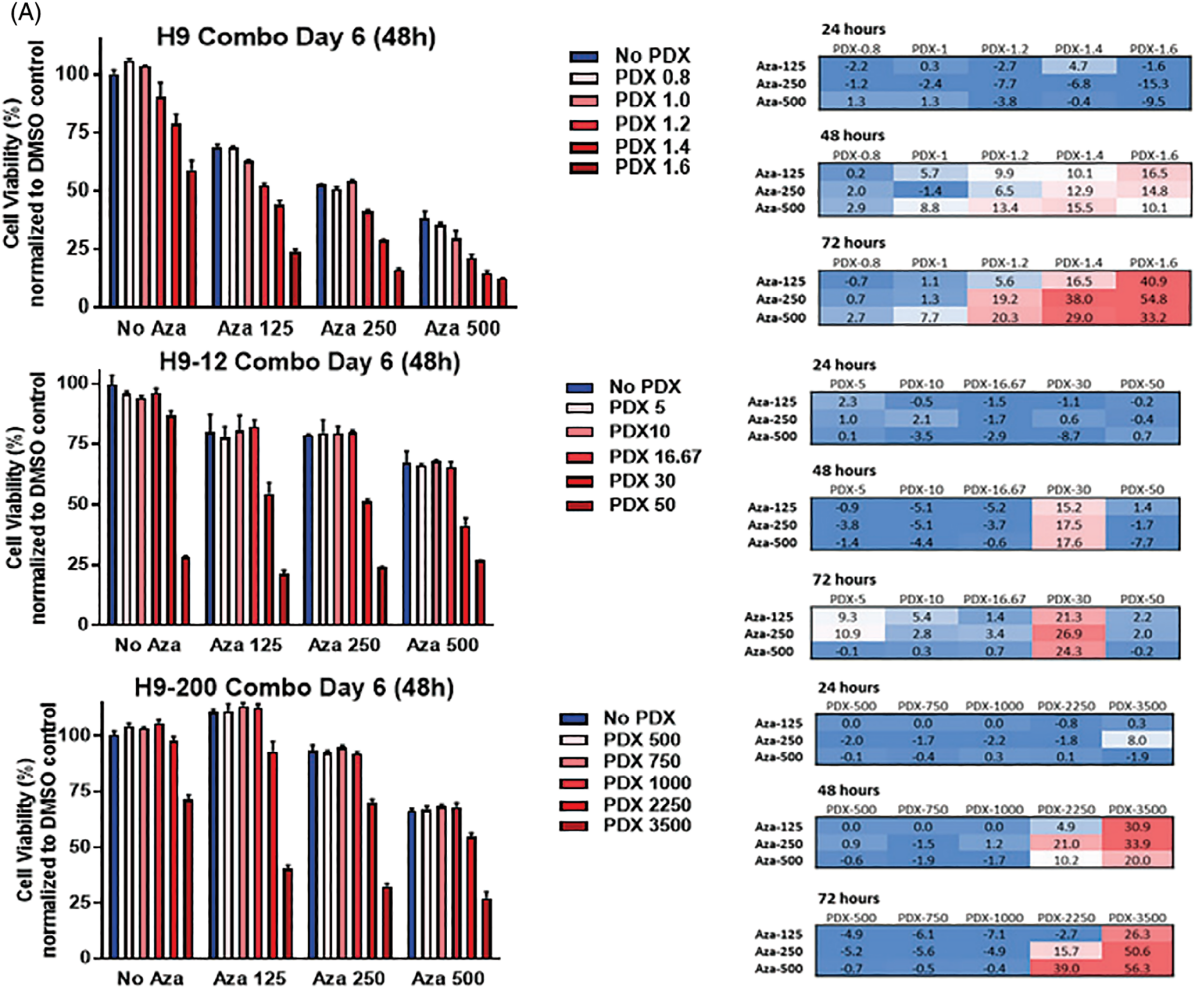
Azacitidine (AZA) and decitabine (DAC) synergize with pralatrexate (PDX) in H9, H‐12, and H9‐200 cells. A, AZA and B, DAC pretreatment sensitizes H9‐12 and H9‐200 cells to PDX. Sequential exposure to increase concentration of hypometylating agent decreases resistance to PDX. Error bars represent the SD of three or more separated experiments. Excess Over Bliss (EOB) values represent average of three independent experiments. AZA, DAC, PDX. Concentration values are in nM [Color figure can be viewed at wileyonlinelibrary.com]

As noted above, all three cell lines were equally sensitive to the hypomethylating agents, and exposure to AZA induced an increase in the *RFC* mRNA level in parental, H9‐12, and H9‐200 cell lines. To explore this further, H9, H9‐12,and H9‐200 were continuously exposed to increasing concentration of AZA and decitabine for 96 hours and then reanalyzed for their sensitivity to PDX. As shown in Figure 5A,B, an increase in concentration and time dependent PDX sensitivity of H9 and H9‐200 cells was observed after AZA and decitabine pretreatment, with a minimal effect observed in H9‐12 cells, expressing high levels of *DHFR*. Increased sensitivity to PDX in H9‐200 correlates with increased exposure to 5‐AZA and DAC, indicating that the resulting resensitization to PDX was due, at least in part, to the hypomethylating agent pretreatment.

### 3.6 Gene expression profiling of H9, H9‐12, and H9‐200 reveals two patterns of modulation

To gain deeper insight into the mechanisms of action and the molecular pathways affected by MTX and PDX, gene expression profiles (GEP) of parental and resistant T‐cell lymphoma lines (H9, H9‐12, and H9‐200) exposed to single agents drugs were performed. Unsupervised hierarchical clustering and PCA revealed a clear distinction and close distribution based on cell types and treatment, indicating the consistency of the experiments as well as the specificity of treatment effects (Supporting information Figure 4).

**FIGURE 6:**
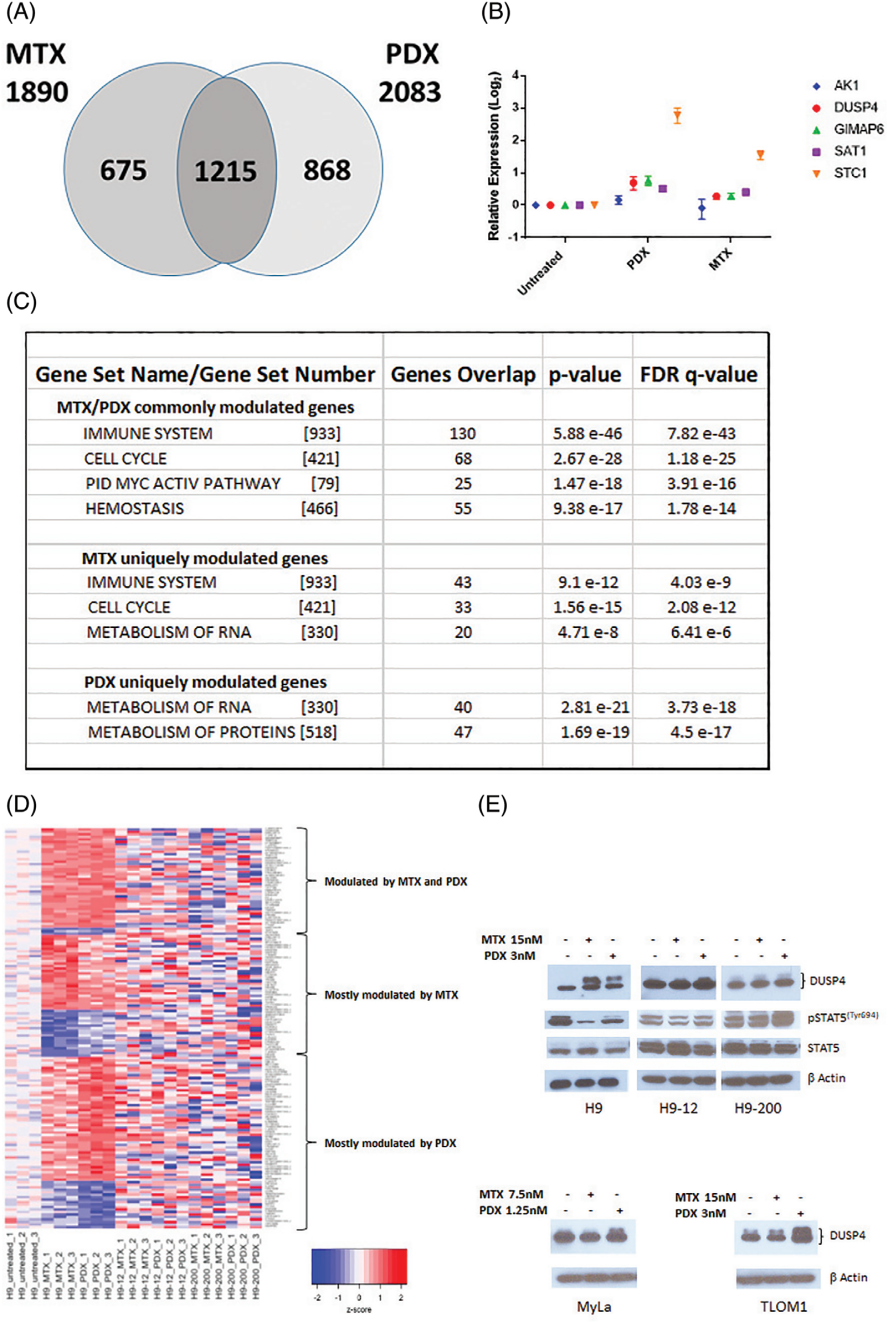
Methotrexate (MTX) and pralatrexate (PDX) modulate unique set of genes. A, The Venn diagram shows the relationship among genes included in the two signatures (adjusted *P* value ≤.05). The expression of 675 and 868 genes, uniquely modulated by each agent treatment. Number of genes modulated by both agents are also shown. B, Validation of the gene expression profiles (GEP) analysis performed by reverse transcription polymerase chain reaction (RT‐qPCR) using five selected genes (*AK1*, dual specificity phosphatase four [*DUSP4*], *GIMAP6*, *SAT1* and *STC1*). C, Canonical pathways commonly and uniquely affected by MTX and PDX. D, Supervised analysis of TCL lines based of the expression of genes modulated by each single agent (adjusted *P* value .05, Log 2‐fold change value ≥0.5). Commonly and uniquely gene sets modulated by MTX and PDX are indicated. E, Western blot analysis of H9, H9‐12, H9‐200 (DUSP4, phospho‐STAT5 and STAT5), MyLa and TLOM1 (DUSP4) cells exposed 24 hours to MTX and PDX [Color figure can be viewed at wileyonlinelibrary.com]

GEP on parental H9 cells treated with MTX or PDX revealed that 1890 genes were modulated by MTX vs 2083 genes modulated by PDX. The relationship among the GEP are shown in Figure 6A. The number of genes modulated in common by PDX and MTX was 1215 genes; 675 of the 1890 MTX‐modulated genes were unique to MTX only, while 860 of the 2083 genes modulated by PDX were unique to PDX. Validation of gene expression analysis was performed by RT‐qPCR using five selected genes (*AK1*, *DUSP4*, *GIMAP6*, *SAT1*, *STC1*), and successfully confirmed the genes found to be up‐regulated in the differential expression analysis (Figure 6B).

Gene set enrichment analysis on the common and unique gene sets identified four canonical pathways (immune system, cell cycle, MYC activation pathway and hemostasis), affected by exposure to both MTX and PDX. An additional 43 and 33 genes that belong to the immune system and cell cycle were affected by exposure to MTX but not PDX. In contrast, exposure to PDX but not MTX affected 47 genes that belong to the metabolism of proteins canonical pathway. Lastly, exposure to both single agents affected the metabolism of RNA pathway but by modulating different (nonoverlapping) set of genes, 20 in case of MTX, 40 for PDX (Figure 6C). A supervised analysis of the genes whose expression is modulated (adjusted *P* value .05, absolute Log 2‐fold change 0.5, Supporting information Tables 1 and 2) by each single agent is shown in Figure 6D. Three patterns of modulation were identified: (a) genes whose expression is modified by both agents, (b) genes whose expression is more substantially modified by MTX than PDX, and (c) genes whose expression is more substantially modified by PDX than MTX. Interestingly, none of the patterns are seen in the two resistant cell lines H9‐12 and H9‐200 following exposure to MTX or PDX.

The DUSP4 was among the genes whose expression was found upregulated after exposure to MTX or PDX (Figure 6B,C). As shown in Figure 6E, increase in DUSP4 protein level was also observed, when H9 and MyLa, two CTCL derived cell lines and TLOM1, an adult T‐cell leukemia derived cell line, were exposed to PDX. DUSP4 has been showed to regulate STAT5 stability in T cells^28^ and suppress CD4 T cell proliferation through regulation of STAT5 phosphorylation and IL‐2 signaling.^29^ As shown in Figure 6E a decrease in STAT5 phosphorylation was observed in parental H9 but not in H9‐12 and H9‐200 resistant cells. Interestingly, the decrease in STAT5 phosphorylation correlates with a corresponding increase in DUSP4 expression.

## 4 DISCUSSION

**FIGURE 7:**
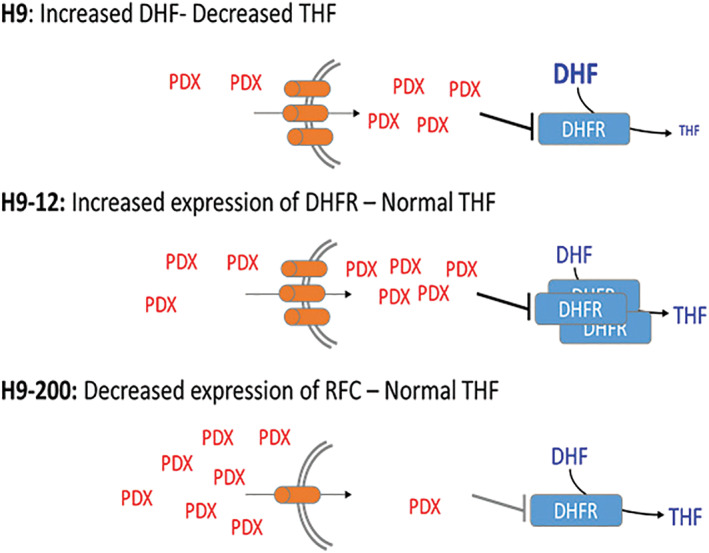
Proposed models of pralatrexate (PDX) resistance in H9‐12 and H9‐200 cell lines. Effect of dihydrofolate reductase (*DHFR*) and reduced folate carrier *(RFC)* deregulated expression in H9‐12 and H9‐200 and models of PDX resistance [Color figure can be viewed at wileyonlinelibrary.com]

The development of drug resistant cell lines can represent a valuable tool in understanding mechanisms of intrinsic and acquired drug resistance, and can provide insights in mechanism of action.^30^, ^31^ Resistance to antifolates has been ascribed to several mechanisms, including increased levels of the target through gene amplification, or genes involved in drug elimination, silencing of internalizing transporters, encompassing transport of the drug, and down‐regulation of genes involved in enhancing its intracellular cytotoxicity.^14^, ^20^, ^25^, ^26^, ^32^, ^33^ We demonstrated herein that in two PDX resistant cell lines, H9‐12 and H9‐200, *DHFR* gene amplification and reduced *RFC1* mRNA expression represent the two major mechanisms resulting in resistance to MTX and PDX, respectively (Figure 7). Pretreatment with hypomethylating agents as AZA and decitabine appear to mitigate some of the observed resistance, although the pretreatment has a more robust effect on H9‐200 cells than the H9‐12 cells. This suggests that the hypomethylating agent is able to overcome the mechanisms of resistance related to *RFC* down‐regulation, and not the *DHFR* amplification. It is possible in fact that the elevated *DHFR* expression in the H9‐12 cell line could be worsened by exposure to the hypomethylating agent, resulting in a further increase in RNA transcriptional levels and increased expression of the *DHFR* gene.

Although the GEP of parental and resistant cell lines after PDX and MTX exposure revealed perturbation of common pathways, the number and type of differentially expressed genes within the common pathways diverge, suggesting that there are substantial differences between these drugs. The notion that PDX has a different mechanism of action making it noncross resistant to MTX has been supported by clinical data, and the observation that leucovorin does not nullify all of its cytotoxic effects.^5^ In this instance, the comparison of GEP analysis of parental and resistant cell lines, exposed to MTX and PDX, could provide mechanistic insights into the differential effects of these drugs, as well as possible mechanism of action, in T‐cell lymphoma.

The identification of *DUSP4* as one of the genes whose expression is increased in H9, MyLa and TLOM1 cells following exposure to PDX is also an example of how we could use this information to generate hypotheses around mechanism. DUSP4 is a member of the dual specificity protein phosphatatse subfamily and DUSPs have been implicated in T‐cell lymphomagenesis. These phosphatases negatively regulate members of the mitogen‐activated kinase superfamily involved in cellular proliferation and differentiation.

Reduced expression of DUSP4 has been identified as an adverse feature in different tumor types and cancer derived cell lines^34^, ^35^, ^36^, ^37^ and found to be epigenetically silenced in 75% of cases of diffuse B cell lymphoma (DLBCL). Moreover, a lack of DUSP4 was a negative prognostic factor in three independent cohorts of DLBCL patients.^38^ An increase in DUSP4 mediated by PDX would theoretically counterbalance the adverse pathogenetic implications of reduced DUSP4.

These findings are further supported by our observation of STAT5 decreased phosphorylation in H9 but not in H9‐12 and H9‐200 exposed to PDX. DUSP4 has shown to regulates STAT5 phosphorylation,^28^ and recent findings show that STAT5 activation drives PTCL,^39^ while its inhibition induces apoptosis in PTCL.^40^ Pimozide, an FDA‐approved neuroleptic agent, shown to be a STAT5 inhibitor, demonstrated a concentration dependent reduction in STAT5 activity and in the number of viable cells in PTCL lines.^40^

Further studies are underway to explore whether the reduction in STAT5 phosphorylation is the result of DUSP4 induced expression by PDX.

In summary, our PDX resistant cell line models represent useful paradigms (a) to elucidate the molecular mechanisms of action of PDX, (b) to clarify the mechanisms underlying clinical resistance to the drug, and (c) to evaluate new therapeutic strategies aimed at overcome drug resistance.

## CONFLICT OF INTEREST

O. O'. C. is a member of the Data Safety Monitoring Board at Celgene. He also receives research support from Celgene. Receives research support from Spectrum Pharmaceuticals. E. M. consulting for Spectrum Pharmaceuticals, Mundipharma. F. M. receives support from Seattle Genetics.

## Supporting information


**Supporting information Figure 1** H9‐12 and H9‐200 retain resistance to PDX and MTX. (A) Growth inhibition curves following 24, 48 and 72 hours exposure of H9‐200 cells up to a 10.000 nM of pralatrexate (PDX). (B) Growth inhibition curves following 24, 48 and 72 hours exposure of H9‐12 (blue) and H9‐200 (red) cells, grown for a month in absence of drug, to increased PDX or methotrexate (MTX) concentration. IC_50_ values given in nanomolar concentrations for each time point. Error bars represent the SD of three or more separated experiments.

**Supporting information Figure 2** H9, H9‐12 and H9‐200 similar sensitivity to bortezomib, doxorubicin and gemcitabine. Growth inhibition curves following 24 (blue), 48 (red) and 72 (green) h exposure to increased bortezomib, doxorubicin and gemcitabine concentration. IC_50_ values given in nanomolar concentrations for each time point. Error bars represent the SD of three or more separated experiments.

**Supporting information Figure 3** Western blot analysis of transcription factor expression in parental and resistant cell lines. Sp1, Sp3, JUN and USF1 protein levels in H9, H9‐12 and H9‐200 cells as determined by western blot analysis.

**Supporting information Figure 4** Unsupervised analyses of GEP of T cell lymphoma lines exposed to MTX and PDX. (A) Unsupervised hierarchical clustering divided the samples according to cell type and within each cell type to drug treatment. In the matrix, each column represents a sample and each row represents a gene. The color scale bar shows the relative gene expression changes normalized by the SD (0 is the mean expression level of a given gene). (B) Principle component analysis (PCA) showed a clear distinction based on cell types and treatment.

**Supporting information Table 1** (Methotrexate)
Supporting information Table 2 (Pralatrexate)

## Data Availability

The data that supports the findings of this study are available in the supplementary material of this article.
