# Supplementary figures and images for: Generation of pralatrexate resistant T‐cell lymphoma lines reveals two patterns of acquired drug resistance that is overcome with epigenetic modifiers
Source: Genes Chromosomes Cancer. 2020 Jul 30;59(11):639–51. doi: 10.1002/gcc.22884 (PMC7540375; doi:10.1002/gcc.22884)

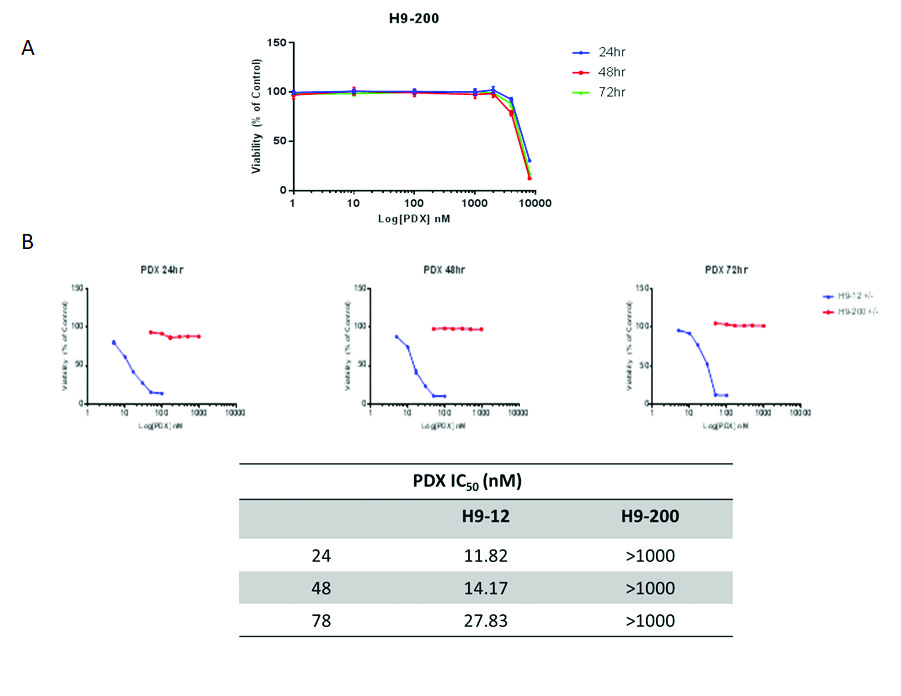

Supplement: Supplementary file 1 — Supporting information Figure 1 H9‐12 and H9‐200 retain resistance to PDX and MTX. (A) Growth inhibition curves following 24, 48 and 72 hours exposure of H9‐200 cells up to a 10.000 nM of pralatrexate (PDX). (B) Growth inhibition curves following 24, 48 and 72 hours exposure of H9‐12 (blue) and H9‐200 (red) cells, grown for a month in absence of drug, to increased PDX or methotrexate (MTX) concentration. IC50 values given in nanomolar concentrations for each time point. Error bars represent the SD of three or more separated experiments. [file GCC-59-639-s001.jpg]

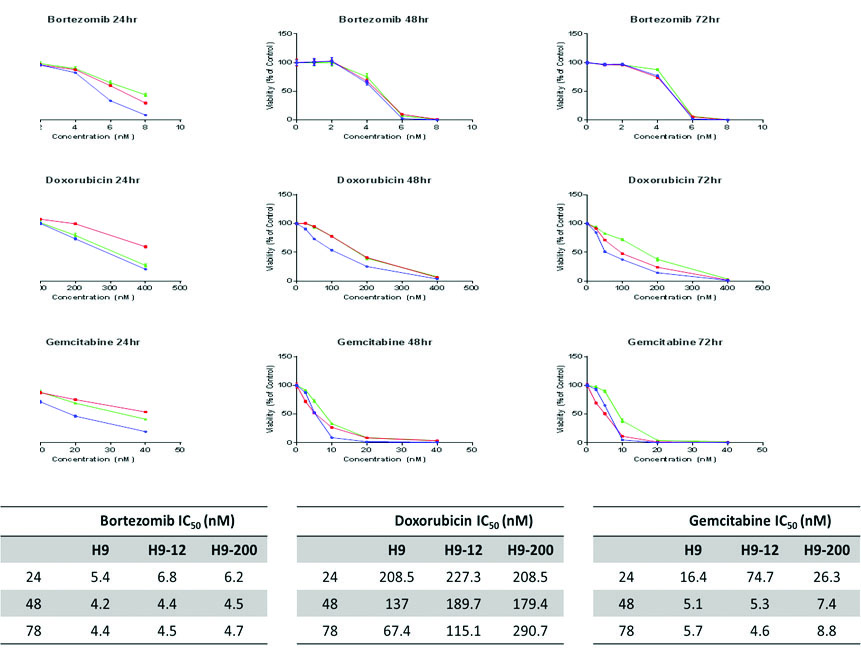

Supplement: Supplementary file 2 — Supporting information Figure 2 H9, H9‐12 and H9‐200 similar sensitivity to bortezomib, doxorubicin and gemcitabine. Growth inhibition curves following 24 (blue), 48 (red) and 72 (green) h exposure to increased bortezomib, doxorubicin and gemcitabine concentration. IC50 values given in nanomolar concentrations for each time point. Error bars represent the SD of three or more separated experiments. [file GCC-59-639-s002.jpg]

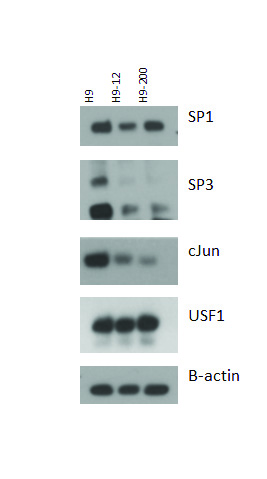

Supplement: Supplementary file 3 — Supporting information Figure 3 Western blot analysis of transcription factor expression in parental and resistant cell lines. Sp1, Sp3, JUN and USF1 protein levels in H9, H9‐12 and H9‐200 cells as determined by western blot analysis. [file GCC-59-639-s003.jpg]

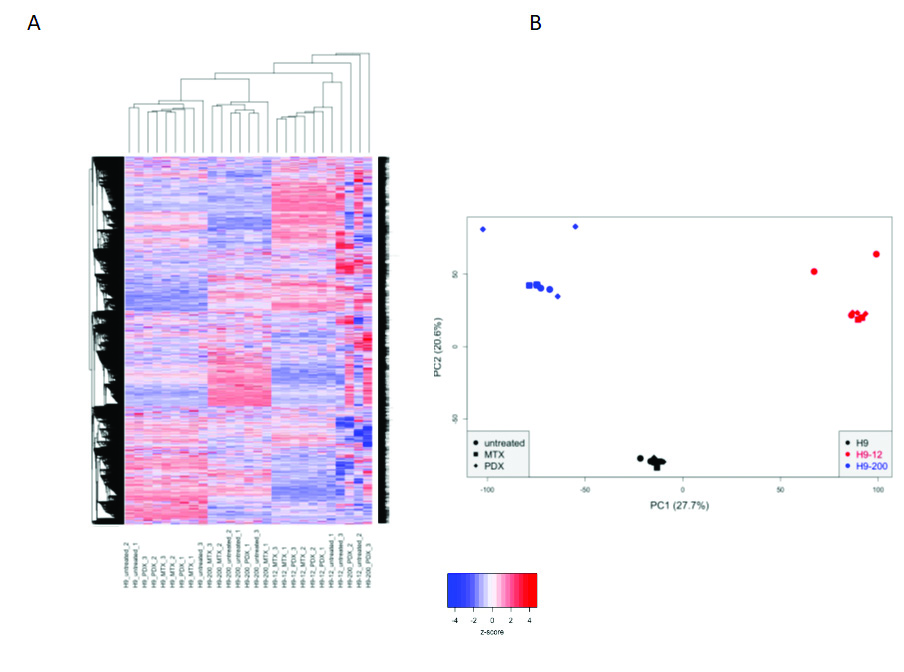

Supplement: Supplementary file 4 — Supporting information Figure 4 Unsupervised analyses of GEP of T cell lymphoma lines exposed to MTX and PDX. (A) Unsupervised hierarchical clustering divided the samples according to cell type and within each cell type to drug treatment. In the matrix, each column represents a sample and each row represents a gene. The color scale bar shows the relative gene expression changes normalized by the SD (0 is the mean expression level of a given gene). (B) Principle component analysis (PCA) showed a clear distinction based on cell types and treatment. [file GCC-59-639-s004.jpg]
