# Supplementary material for: Generation of pralatrexate resistant T‐cell lymphoma lines reveals two patterns of acquired drug resistance that is overcome with epigenetic modifiers
Source: Genes Chromosomes Cancer. 2020 Jul 30;59(11):639–51. doi: 10.1002/gcc.22884 (PMC7540375; doi:10.1002/gcc.22884)
Supplement: Supplementary file 5 — Supporting information Table 1 (Methotrexate) Supporting information Table 2 (Pralatrexate) [file GCC-59-639-s005.docx]

Supporting information Table 1 (Methotrexate)

| **Gene ID** | **log2 fold change** | ***P* value** | **Modulation** |
| --- | --- | --- | --- |
| MFSD2A | 3.93017827 | 0.005969931 | **↑** |
| C15orf52 | 3.80311653 | 0.008528421 | **↑** |
| ACTL9 | 3.126244304 | 0.048553649 | **↑** |
| FAM157B | 3.125989014 | 0.047871201 | **↑** |
| DSCAML1 | 2.475892505 | 0.005003358 | **↑** |
| LOC100506827 | 2.465458712 | 0.026874539 | **↑** |
| LOC100128242 | 1.815831317 | 0.00508375 | **↑** |
| EPHA2 | 1.740356635 | 0.041082604 | **↑** |
| GPR84 | 1.622187794 | 0.049457526 | **↑** |
| ADAM33 | 1.604981913 | 0.005458974 | **↑** |
| C15orf26 | 1.494561368 | 0.049686238 | **↑** |
| IL1RL1 | 1.357336567 | 0.0000198 | **↑** |
| TMEM86A | 1.112202384 | 0.022667049 | **↑** |
| ACRBP | 1.108713504 | 0.0000847 | **↑** |
| TMEM213 | 1.077930925 | 0.000000665 | **↑** |
| OPRL1 | 1.064043867 | 0.003177635 | **↑** |
| TNNT1 | 1.03621556 | 0.048508053 | **↑** |
| LOC100507347 | 1.02838123 | 0.002876882 | **↑** |
| GIMAP4 | 0.950455023 | 0.000000162 | **↑** |
| LOC399715 | 0.93261811 | 0.001089812 | **↑** |
| FCGBP | 0.908566738 | 1.79E-31 | **↑** |
| LOC100505695 | 0.876809872 | 0.047258831 | **↑** |
| CD72 | 0.873335844 | 0.021339629 | **↑** |
| PRDM8 | 0.860364265 | 4.18E-17 | **↑** |
| LINC00324 | 0.84525137 | 0.012029122 | **↑** |
| LOC100128420 | 0.81004279 | 0.01306903 | **↑** |
| MGC12916 | 0.795528416 | 0.000104907 | **↑** |
| CASS4 | 0.788204525 | 0.000182581 | **↑** |
| GRIP2 | 0.758843763 | 8.72E-38 | **↑** |
| HERC2P7 | 0.737954759 | 0.001621333 | **↑** |
| CEACAM16 | 0.736649042 | 0.010551439 | **↑** |
| ABCA4 | 0.734305207 | 0.000588597 | **↑** |
| ZNF599 | 0.7208055 | 0.045019495 | **↑** |
| CD300A | 0.717313054 | 0.043369633 | **↑** |
| GIMAP6 | 0.710983698 | 8.34E-34 | **↑** |
| TP53INP1 | 0.709804167 | 0.038573225 | **↑** |
| C2orf48 | 0.701369727 | 0.008220482 | **↑** |
| NPTX1 | 0.689561952 | 0.00000264 | **↑** |
| PITPNM3 | 0.68529992 | 0.046724996 | **↑** |
| SCARF2 | 0.634911859 | 0.044804038 | **↑** |
| LGALS9C | 0.619835822 | 0.001113918 | **↑** |
| DUSP4 | 0.617597905 | 0.000247312 | **↑** |
| APLNR | 0.600755527 | 0.004085863 | **↑** |
| ITGB4 | 0.590987837 | 0.000160284 | **↑** |
| LY9 | 0.587928048 | 0.000049 | **↑** |
| SOAT2 | 0.587711726 | 0.002447139 | **↑** |
| PGLYRP4 | 0.587249044 | 0.0000819 | **↑** |
| MEI1 | 0.569515843 | 0.017344683 | **↑** |
| MAFF | 0.567342839 | 0.0000166 | **↑** |
| STYK1 | 0.563742271 | 3.04E-11 | **↑** |
| MYO15B | 0.562381023 | 0.000735745 | **↑** |
| MAPK11 | 0.552287198 | 0.0000682 | **↑** |
| TGFB3 | 0.544085947 | 0.020533964 | **↑** |
| NOS3 | 0.537494553 | 0.004571507 | **↑** |
| ATP2C2 | 0.535188479 | 0.005162124 | **↑** |
| ABHD4 | 0.533632047 | 0.000489543 | **↑** |
| TOX2 | 0.530988683 | 1.68E-17 | **↑** |
| RDM1 | 0.52583718 | 0.015025691 | **↑** |
| LOC100507164 | 0.525691829 | 0.000000114 | **↑** |
| LOC100506354 | 0.522382773 | 0.03403283 | **↑** |
| MMP9 | 0.520698565 | 1.42E-15 | **↑** |
| ENG | 0.517914445 | 0.020917112 | **↑** |
| SPRY4 | 0.516405521 | 8.35E-09 | **↑** |
| CKLF | 0.516027621 | 0.046862608 | **↑** |
| ATG16L2 | 0.513334801 | 7.11E-11 | **↑** |
| LOC100133445 | 0.51317299 | 0.002457196 | **↑** |
| RASGEF1A | 0.509675989 | 0.041680096 | **↑** |
| LOC100507173 | 0.509360388 | 0.010016589 | **↑** |
| DUSP6 | 0.502431547 | 9.26E-85 | **↑** |
| SAT1 | 0.501289842 | 4.25E-08 | **↑** |
| LOC100506748 | -0.50777299 | 0.021007728 | **↓** |
| LOC100190986 | -0.518610302 | 0.001372598 | **↓** |
| KIAA1984 | -0.533975106 | 0.029534282 | **↓** |
| HIST2H2BE | -0.544268841 | 0.004027108 | **↓** |
| OXTR | -0.554406884 | 0.023038437 | **↓** |
| C10orf103 | -0.57825528 | 0.026146796 | **↓** |
| SLC14A2 | -0.588874325 | 0.038702576 | **↓** |
| PDF | -0.698882161 | 0.045107381 | **↓** |
| TSSK2 | -0.732712034 | 0.019860061 | **↓** |
| CARD9 | -0.733366194 | 0.045827906 | **↓** |
| CHAC1 | -0.735529812 | 0.021687145 | **↓** |
| LOC100505873 | -0.863812645 | 0.021410238 | **↓** |
| CAV1 | -0.885537265 | 0.045244861 | **↓** |
| CLUL1 | -1.019460322 | 0.045657533 | **↓** |
| CDKL4 | -1.151076293 | 0.042736008 | **↓** |
| GUSBP3 | -1.236024155 | 0.001672903 | **↓** |
| SLC2A7 | -1.434798766 | 0.041187346 | **↓** |
| ZNF578 | -1.738416437 | 0.032892521 | **↓** |
| DCAF4L1 | -1.893374363 | 0.032835793 | **↓** |
| PCDHGA12 | -3.875390601 | 0.031372947 | **↓** |
| VSNL1 | -3.99146846 | 0.02328621 | **↓** |
| SGK3 | -4.108566785 | 0.017164502 | **↓** |

**Supporting information Table 1.** List of genes modulated in H9 cells after 24 h exposure to methotrexate (MTX) (15 nM). Absolute Log2 fold change 0.5, *P*-value <0.05). ↑ upreglation, ↓ downregulation.

Supporting information Table 2 (Pralatrexate)

| **Gene ID** | **log2 fold change** | ***P* value** | **Modulation** |
| --- | --- | --- | --- |
| STC1 | 3.552458038 | 0.045051634 | **↑** |
| FP588 | 3.550015382 | 0.048325042 | **↑** |
| OR7D2 | 3.275197756 | 0.033085884 | **↑** |
| GJB2 | 3.167438209 | 0.041234468 | **↑** |
| SEC16B | 2.7591917 | 0.038686138 | **↑** |
| AKR1C1 | 2.681173822 | 0.047033505 | **↑** |
| DSCAML1 | 2.412211185 | 0.005685482 | **↑** |
| LOC151534 | 2.328426662 | 0.041383751 | **↑** |
| MRPL23-AS1 | 1.816437802 | 0.001737871 | **↑** |
| LOC100653018 | 1.809407067 | 0.008959213 | **↑** |
| PIPOX | 1.80151494 | 0.005589384 | **↑** |
| SLC16A8 | 1.769365412 | 0.031558864 | **↑** |
| C15orf26 | 1.748947605 | 0.014974769 | **↑** |
| TP53INP1 | 1.614035748 | 4.97E-08 | **↑** |
| ANGPT4 | 1.551896025 | 0.049149211 | **↑** |
| ADAM33 | 1.471214825 | 0.013676994 | **↑** |
| LOC100507520 | 1.290816896 | 0.011358089 | **↑** |
| TMEM213 | 1.267470348 | 8.27E-10 | **↑** |
| GIMAP4 | 1.266358618 | 1.39E-14 | **↑** |
| MT1H | 1.262478889 | 0.024495254 | **↑** |
| RAB41 | 1.242543243 | 0.047125047 | **↑** |
| NR4A2 | 1.236463094 | 0.014634015 | **↑** |
| FCGBP | 1.21256358 | 5.25E-60 | **↑** |
| C17orf108 | 1.179916991 | 0.013774401 | **↑** |
| MGC12916 | 1.146398626 | 1.79E-09 | **↑** |
| IL1RL1 | 1.13042188 | 0.000585977 | **↑** |
| OPRL1 | 1.125030387 | 0.001699881 | **↑** |
| TMEM86A | 1.064606043 | 0.034883727 | **↑** |
| LOC100131289 | 1.039512478 | 0.017537032 | **↑** |
| MIR25 | 1.008491548 | 0.007580257 | **↑** |
| LOC399715 | 0.979788939 | 0.000781317 | **↑** |
| GIMAP6 | 0.971898637 | 6.85E-65 | **↑** |
| PRDM8 | 0.887835966 | 5.6E-17 | **↑** |
| LOC100507347 | 0.886718807 | 0.011620818 | **↑** |
| ABCA4 | 0.873213725 | 0.0000244 | **↑** |
| MYCBPAP | 0.871489379 | 0.032746009 | **↑** |
| NELL2 | 0.857867471 | 0.0000933 | **↑** |
| LINC00324 | 0.828134377 | 0.013405117 | **↑** |
| LOC100506425 | 0.828037467 | 0.038164363 | **↑** |
| LOC100128420 | 0.816399112 | 0.011390436 | **↑** |
| CASS4 | 0.804584352 | 0.000146484 | **↑** |
| TBX6 | 0.796818662 | 0.010033267 | **↑** |
| LOC100130950 | 0.778053284 | 0.024337513 | **↑** |
| CDH23 | 0.775811137 | 0.041741823 | **↑** |
| RDM1 | 0.748170415 | 0.000305065 | **↑** |
| FAM43A | 0.747746971 | 0.008575391 | **↑** |
| FOXB1 | 0.733457752 | 0.021574625 | **↑** |
| PITPNM3 | 0.72558763 | 0.034698905 | **↑** |
| MAPK11 | 0.721630424 | 7.86E-08 | **↑** |
| MAML2 | 0.719500935 | 1.75E-08 | **↑** |
| DUSP15 | 0.709621825 | 0.02247624 | **↑** |
| ACRBP | 0.692964492 | 0.021369569 | **↑** |
| MYO15B | 0.673777393 | 0.0000488 | **↑** |
| CEACAM16 | 0.670037487 | 0.02979213 | **↑** |
| LOC100130231 | 0.652177577 | 0.00000179 | **↑** |
| CGNL1 | 0.65092682 | 0.00264769 | **↑** |
| PGLYRP4 | 0.649691596 | 0.00000869 | **↑** |
| EXD3 | 0.63696699 | 0.029512568 | **↑** |
| SCARF2 | 0.636962997 | 0.044846167 | **↑** |
| GRIP2 | 0.636326426 | 2.45E-27 | **↑** |
| LOC100132832 | 0.635313794 | 0.03633678 | **↑** |
| LY9 | 0.633500126 | 0.00000896 | **↑** |
| C2orf48 | 0.62766487 | 0.022211526 | **↑** |
| NPTX1 | 0.614004111 | 0.0000279 | **↑** |
| MPZ | 0.610361459 | 3.44E-09 | **↑** |
| RASGEF1A | 0.609472739 | 0.012052441 | **↑** |
| ATG16L2 | 0.60880954 | 3.13E-15 | **↑** |
| OBFC2A | 0.607782471 | 2.64E-48 | **↑** |
| CD2 | 0.607392735 | 0.047337066 | **↑** |
| SAT1 | 0.604778551 | 1.89E-11 | **↑** |
| C16orf93 | 0.601114228 | 0.000161437 | **↑** |
| MMP9 | 0.592074567 | 1.14E-17 | **↑** |
| DUSP4 | 0.578184631 | 0.000320588 | **↑** |
| GIMAP1 | 0.573484082 | 0.00000693 | **↑** |
| NOG | 0.563532597 | 0.002285546 | **↑** |
| LOC100507164 | 0.563257891 | 6.09E-09 | **↑** |
| AIFM2 | 0.561990389 | 0.010982389 | **↑** |
| EVI2A | 0.559709248 | 6.69E-11 | **↑** |
| C5orf41 | 0.558045456 | 0.040667568 | **↑** |
| HERC2P7 | 0.553515611 | 0.021158273 | **↑** |
| CCNG2 | 0.545497966 | 1.84E-09 | **↑** |
| LOC100270710 | 0.542104354 | 0.006421367 | **↑** |
| PSEN2 | 0.526439026 | 8.19E-09 | **↑** |
| LOC100132731 | 0.523813709 | 0.045422623 | **↑** |
| LOC100133445 | 0.522041426 | 0.002293529 | **↑** |
| TOX2 | 0.515923012 | 8.52E-15 | **↑** |
| LOC100652789 | 0.513106145 | 0.0000124 | **↑** |
| FAM109A | 0.512423009 | 0.016544366 | **↑** |
| LOC100506159 | 0.512180215 | 0.038628294 | **↑** |
| AK1 | 0.511149942 | 0.00121347 | **↑** |
| FAM203A | 0.509915191 | 0.008688201 | **↑** |
| KIAA1984 | -0.512911523 | 0.034940486 | **↓** |
| FOSL2 | -0.524056295 | 0.017724164 | **↓** |
| SOCS3 | -0.552578609 | 3.37E-15 | **↓** |
| ARL6 | -0.562693035 | 0.020663868 | **↓** |
| RNF207 | -0.60097812 | 0.001834868 | **↓** |
| PDF | -0.788273275 | 0.019195124 | **↓** |
| JPH3 | -0.821613619 | 0.039172428 | **↓** |
| HIST2H2BF | -0.861590741 | 0.033804939 | **↓** |
| KCNAB1 | -0.926319588 | 0.021396975 | **↓** |
| RYR3 | -0.929628342 | 0.023698283 | **↓** |
| 43346 | -0.956820662 | 0.041347654 | **↓** |
| RNU2-2 | -1.519833116 | 0.014138613 | **↓** |
| PFN4 | -1.586299472 | 0.045570135 | **↓** |
| ACY1 | -1.645087064 | 0.021236953 | **↓** |
| SNX24 | -1.878704979 | 0.010673147 | **↓** |
| ZNF578 | -2.001116071 | 0.02104074 | **↓** |
| MYADML2 | -2.187150811 | 0.043101545 | **↓** |
| GRM1 | -2.407290982 | 0.046965006 | **↓** |
| C9orf152 | -2.887719328 | 0.034536913 | **↓** |
| GGT2 | -3.389422301 | 0.038765511 | **↓** |
| LOC100506431 | -3.693376917 | 0.018034716 | **↓** |
| ZNF781 | -3.713054493 | 0.046360745 | **↓** |
| GPR45 | -3.817632145 | 0.011586597 | **↓** |

**Supporting information Table 2.** List of genes modulated in H9 cells after 24 h exposure to pralatrexate (PDX) (3 nM). Absolute Log2 fold change 0.5, *P*-value <0.05). ↑ upreglation, ↓ downregulation.
